# Low-Cost High-Performance MRI

**DOI:** 10.1038/srep15177

**Published:** 2015-10-15

**Authors:** Mathieu Sarracanie, Cristen D. LaPierre, Najat Salameh, David E. J. Waddington, Thomas Witzel, Matthew S. Rosen

**Affiliations:** 1 MGH/A.A. Martinos Center for Biomedical Imaging, 149 13th St, Suite 2301, Charlestown MA 02129, USA; 2 Department of Physics, Harvard University, 17 Oxford St, Cambridge, MA 02138, USA; 3 Institute of Physics of Biological Systems, Ecole Polytechnique Fédérale de Lausanne, CH-1015 Lausanne, Switzerland; 4 School of Physics, University of Sydney, Physics Rd, Sydney NSW 2006, Australia; 5 Harvard Medical School, 25 Shattuck St, Boston, MA 02115, USA

## Abstract

Magnetic Resonance Imaging (MRI) is unparalleled in its ability to visualize anatomical structure and function non-invasively with high spatial and temporal resolution. Yet to overcome the low sensitivity inherent in inductive detection of weakly polarized nuclear spins, the vast majority of clinical MRI scanners employ superconducting magnets producing very high magnetic fields. Commonly found at 1.5–3 tesla (T), these powerful magnets are massive and have very strict infrastructure demands that preclude operation in many environments. MRI scanners are costly to purchase, site, and maintain, with the purchase price approaching $1 M per tesla (T) of magnetic field. We present here a remarkably simple, non-cryogenic approach to high-performance human MRI at ultra-low magnetic field, whereby modern under-sampling strategies are combined with fully-refocused dynamic spin control using steady-state free precession techniques. At 6.5 mT (more than 450 times lower than clinical MRI scanners) we demonstrate (2.5 × 3.5 × 8.5) mm^3^ imaging resolution in the living human brain using a simple, open-geometry electromagnet, with 3D image acquisition over the entire brain in 6 minutes. We contend that these practical ultra-low magnetic field implementations of MRI (<10 mT) will complement traditional MRI, providing clinically relevant images and setting new standards for affordable (<$50,000) and robust portable devices.

Magnetic Resonance Imaging (MRI) is a powerful, non-invasive technique for revealing the internal structure and function of the human body with a rich range of biological contrasts. Despite considerable improvements in imaging quality and speed, the underlying technology remains remarkably unchanged compared to the first generation scanners that emerged on the market 30 years ago. The fact that very strong magnetic fields are needed to overcome the intrinsic lack of sensitivity of NMR-based methods continues to dominate scanner construction, and drives both pricing and scanner siting requirements. MRI scanners are built around massive superconducting magnets with a nominal cost of $1 M per tesla of magnetic field. With 1.5 tesla (T) and 3 T scanners in common use, and increasing demand for 7 T, the extreme cost of these devices limits the number of scanners on site and requires hospitals to carefully prioritize patients. Additionally, these massive scanners are strictly confined to the MRI suite within a hospital thus precluding mobile operation in many environments including surgical intervention, triage and primary care suites.

Undeniably, one of the next revolutions in health care will center on cost-effectiveness. Thus the prospect of low-cost (<$50.000) but high-performance MRI systems to complement traditional MRI scanners is compelling. A promising solution is MRI at very low magnetic field where scalable electromagnets become practical. Operation at low magnetic field enables imaging in environments where high magnetic fields would be contraindicated (such as in the presence of nearby ferrous materials), and raises the potential for scanners to be built at significantly reduced total cost, and with open geometry designs that ease patient handling and positioning.

The unique role that very low magnetic field MRI scanners can play in neurocritical care was recognized 30 years ago in the pioneering work of Sepponnen, *et al.*^1^, who explored the clinical validity of brain MRI acquired in a 20 mT scanner located in a hospital emergency department. These early images were acquired at the lowest field strength reported in clinical MRI at that time, and although limited to a single 15 mm slice, were obtained with good contrast in a reasonable four minute acquisition.

In an effort to improve the performance of very low field MRI systems, Macovski and Conolly introduced the concept of pre-polarized MRI (also known as PMRI) in 1993^2^, which employs a strong, inhomogeneous pulsed magnet field to generate increased nuclear polarization, and a second much weaker homogeneous magnetic field for signal detection. This PMRI strategy has been the acquisition strategy for nearly all very low field MRI systems since its introduction. In 2006, PMRI in human subjects with metal implants was reported *in vivo* in human wrists^3^, where a 0.4 T field was used for pre-polarization, and a 54 mT field used for signal detection.

The ultra-low field (ULF) MRI regime is defined^4^ when the magnetic field used for signal detection is below 10 mT. In 2007, PMRI was demonstrated with detection in the ULF regime, orders of magnitude lower than reported in Venook *et al.*^3^, using arrays of very sensitive superconducting quantum interference devices (SQUIDs)^5^ as magnetometers to measure the spatially encoded nuclear spin precession^6^. Pre-polarized cryogenic SQUID-detected ULF MRI has been demonstrated in the human brain as well as in the human hand and wrist by several groups^7^,^8^,^9^,^10^,^11^,^12^,^13^,^14^,^15^. Results from late 2013 demonstrate *in vivo* 2D images of the human brain (pre-polarized to 80 mT) with (2.5 × 1.9) mm^2^ in-plane resolution over a (10 × 10) cm^2^ region of interest and a 100 mm thick slice, acquired in 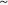
26 minutes^16^. Very recent results from the Los Alamos ULF effort demonstrate 3D images of the human brain (pre-polarized to 100 mT) with (2.1 × 2.4 × 15) mm^3^ resolution (5 slices) in 67 minutes^15^.

Although SQUIDs are the most technically mature of the non-inductive magnetometers used at ULF, several alternative detection technologies have been explored. Optical measurement of nitrogen–vacancy (NV) color centers in diamond^17^,^18^,^19^ form the basis of robust solid-state magnetometers with unmatched magnetic field sensitivity at nanoscale resolutions. As of yet, however, NV-diamond magnetometers do not provide obvious benefits for human scale MRI. Atomic magnetometers (AM) have also been applied to pre-polarized NMR^20^ and MRI^21^,^22^, and improvements in these devices have resulted in a magnetic field sensitivity approaching SQUID performance^23^ without the need for cryogenics. The first attempt at imaging the living human brain with an atomic magnetometer was reported in 2013^24^. In this work, nuclear spins are pre-polarized at 80 mT and detection is performed at 4 mT. Despite the ultra-high sensitivity and dynamic range of the AM magnetometer, the setup as described provides limited 3D coverage and significant improvement in resolution and SNR (Signal-to-Noise Ratio) is needed in order to clearly discern anatomical features, which will inevitably increase the acquisition time.

Independent of which detection technology is used, all pre-polarized ULF MRI suffers from intrinsically long acquisition times, most of which is incompressible, that result from the time needed to generate nuclear polarization. In the present work, we demonstrate fast and efficient brain ULF MRI at 6.5 mT with no pre-polarization nor cryogenics, combining under-sampling strategies with a high performance fully refocused steady-state-based acquisition in a simple, inexpensive system. With a novel inductive single channel detector, we report the fastest 3D MRI of the living human brain in the ULF regime compared to the state-of-the-art as reported in the literature^11^,^12^,^15^,^16^,^24^.

## Results

### Ultra-low field acquisition strategy

High performance imaging at ultra-low magnetic field focuses on significantly reducing acquisition time using fast imaging techniques. Here, fast imaging was enabled using 3D balanced steady state free precession sequences (b-SSFP)^25^. Originally described by Carr in 1958 as a technique for improving the signal-to-noise ratio (SNR) in NMR experiments^26^, b-SSFP was implemented as an efficient acquisition strategy for MRI in 1986 by Oppelt *et al.*^27^, and extensively investigated in the early 2000 s^25^,^28^,^29^,^30^,^31^,^32^. Unlike traditional gradient- and spin-echo techniques, b-SSFP sequences dynamically refocus spin magnetization following measurement, eliminating the extra delays typically used for *T*_2_ decay and *T*_1_ recovery. This considerably reduces acquisition times and provides the highest SNR per unit time of all imaging sequences^25^,^27^. These sequences are very sensitive to the amount of spin dephasing that occurs between consecutive RF pulses (the pulse repetition time, TR), and typical banding artifacts are expected to appear within a range of  ±1/(2*TR) Hz that result from inhomogeneity in the static magnetic field^25^. This sets a strict requirement on the absolute field homogeneity over the field-of-view (FOV), which for operation at 3 T is typically at the sub-PPM level.

**Figure 1:**
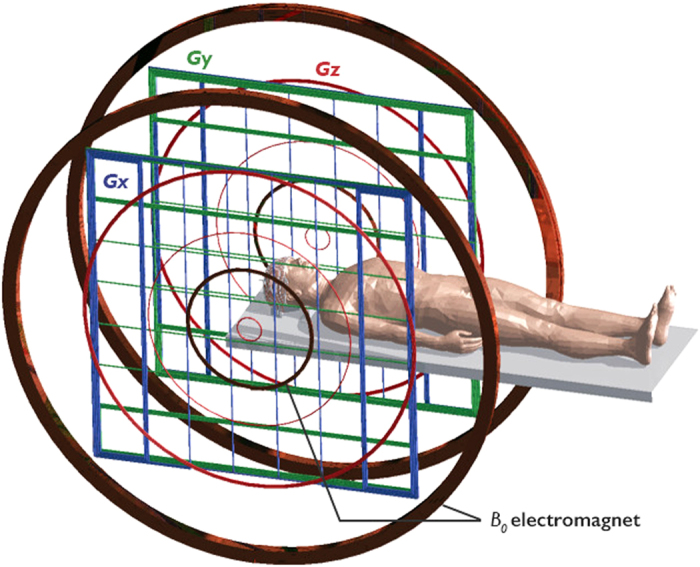
Ultra-low field MRI system. Custom built biplanar 6.5 mT electromagnet with biplanar gradients (Gx, Gy, and Gz). The diameter of the outermost B_0_ coil is 220 cm. The subject lays supine in the scanner and a custom built single channel transmit/receive spiral head coil wound with litz wire for operation at 276 kHz is placed to cradle the head.

In the millitesla regime, however, the fractional homogeneity requirement is three orders of magnitude lower, significantly easing the engineering burden for low-field magnet design. With a current TR = 22.5 ms, our b-SSFP sequence is completely immune to banding artifacts for up to 160 ppm inhomogeneity at 6.5 mT. Furthermore, magnetic susceptibility differences are significantly reduced at ULF, preventing off-resonance b-SSFP artifacts. As a result, provided reasonable magnetic field homogeneity, b-SSFP at very low magnetic field alleviates the necessity of ultra-short TRs and provides good image quality over a large FOV without the need for sophisticated ultrafast gradient power amplifiers. Our 6.5 mT MRI scanner^33^ (Fig. 1) was upgraded for improved 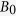 stability^34^, and was used for all the low-field b-SSFP experiments described here.

### RF Coil design

**Figure 2:**
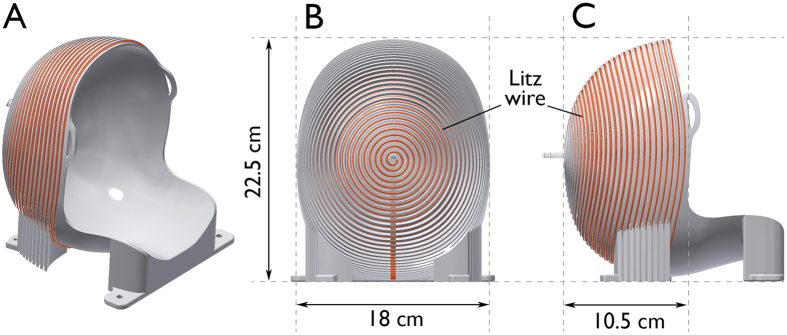
3D renderings of the single channel form-fitting head coil. (**A**) isometric, (**B**) back, and (**C**) side views are shown. The final design was 3D printed on a Fortus 360 mc printer (Stratasys, Eden Prairie, MN, USA) in polycarbonate using fused deposition modeling technology. The 30-turn spiral was wound with Type 1 40/38 Litz wire, parallel resonated to 276 kHz, and capacitively matched to 50 ohms.

The design of inductive detection coils for use in ULF MRI presents a different set of challenges to those present in conventional high-field MRI. In particular, issues of coil resistance and probe bandwidth manifest differently. In conventional MRI, the dominant source of noise is the presence of small currents in the lossy sample (the so-called “body noise” regime) to which a characteristic sample resistance *R*_*S*_ is attributed. Both the sample and the coil contribute to Johnson noise but in practice *R*_*S*_ is much larger than the coil resistance *R*_*C*_ (i.e. *R*_*S*_>>*R*_*C*_), and thus *R*_*C*_ can be neglected in SNR calculations. However, at low field, *R*_*S*_ becomes much smaller and *R*_*C*_ becomes the dominant noise contribution (i.e., the so-called Johnson noise dominated regime). To minimize the coil resistance in a simple design, larger diameter wire or stranded litz wire can be used, but one needs to consider the impact this has on coil bandwidth. Given the maximum imaging gradient strength of ~1 mT/m attainable in our 6.5 mT electromagnet Low Field Imager (LFI), a 20 cm (head-sized) FOV will span a frequency encode bandwidth of ~10 kHz. This sets the minimum bandwidth needed for the detection circuit so as to not significantly convolve the coil response function with the object being imaged. At our Larmor frequency of 276 kHz, this corresponds to a maximum coil *Q* of ~30. A single channel inductive coil for operation at 276 kHz (Fig. 2) was designed and built using 3D printing fused deposition modeling technology and multi-strand litz wire^35^. A 30-turn 3D Archimedean spiral with an aligned turn-to-turn distance of 5.6 mm guided wire placement, thus ensuring that 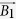 produced by the spiral pattern is everywhere orthogonal to the main magnetic field 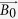. The hemispheric spiral design results in a very homogeneous magnetic field^36^,^37^ over the volume of interest, making it suitable for both RF transmit and receive. The number of turns in the coil was chosen to obtain the inductance needed to achieve the desired *Q*. Litz wire was preferred in this low frequency application due to its lower AC resistance compared to solid copper wire of the same physical size.

### Image reconstruction and processing

MRI images are reconstructed from frequency- and phase-encoded information in the *k*-space formalism^38^,^39^. Previously, we described our use of undersampling strategies to accelerate low-field imaging^34^. We make use of this here by randomly sampling 50% of *k*-space using a variable density Gaussian pattern. The variable density Gaussian sampling pattern emphasizes sampling in the center of *k*-space, where most of the information is located, and randomly skips lines near the edges. The resulting images do not exhibit coherent artifacts, such as typical wrap-around ghosts due to FOV contraction. Missing values in the acquired *k*-space were set to zero. The standard deviation of the sampling pattern as a fraction of the FOV was optimized to preserve adequate high-frequency information. Once reconstructed, the images were apodized and processed using Perona and Malik anisotropic diffusion filtering^40^,^41^ (ADF). ADF is a powerful denoising filter that convolves images of interest with adaptive Gaussian kernels. The Perona and Malik approach works as an iterative multi-scale smoothing and edge detection process that removes noise but prevents image blurring by adjusting filter sharpness as a function of signal intensity gradients.

### *In vivo* brain MRI at 6.5 mT

**Figure 3:**
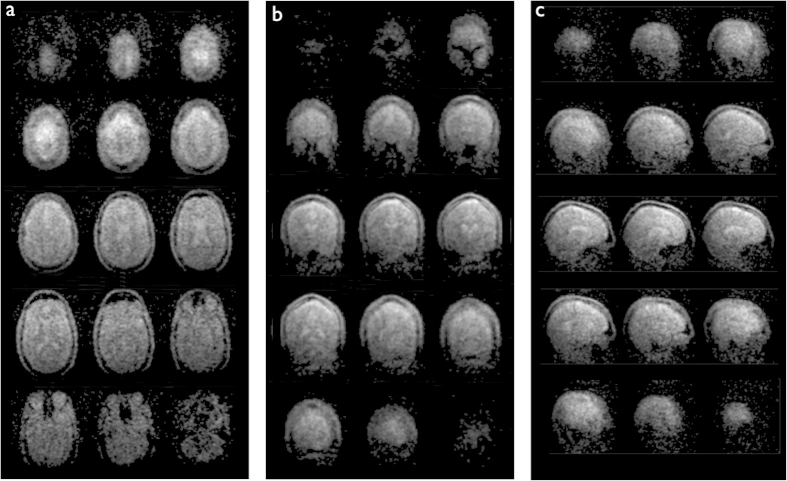
3D images of the living brain acquired in 6 minutes at 6.5 mT in (**a**) axial, (**b**) coronal, and (**c**) sagittal orientation. The corresponding maximum SNRs are a. 15, b. 21, and c. 16. Acquisition matrix: 64 × 75 × 15, voxel size: a. (2.5 × 3.5 × 8.5) mm^3^, b. (2.5 × 3.5 × 11.5) mm^3^, and c. (2.5 × 3.5 × 14.4) mm^3^.

**Figure 4:**
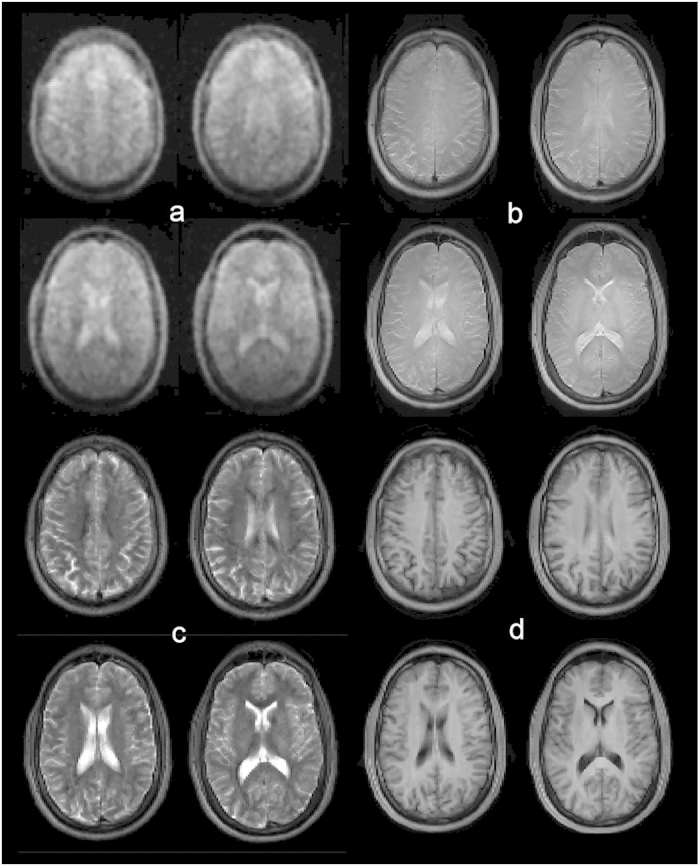
Comparison of single channel ULF MRI to 32-channel high magnetic field scans. (**a**) b-SSFP at 6.5 mT. (**b**–**d**), *PD*, 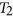, and 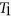 weighted contrast at 3 T, respectively. Most of the anatomic features seen at higher magnetic field can be identified on the ultra-low field scans. At low field, 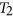 approaches 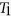, and the resulting image contrast in (**a**) is very similar to *PD*-weighting (**b**).

**Figure 5:**
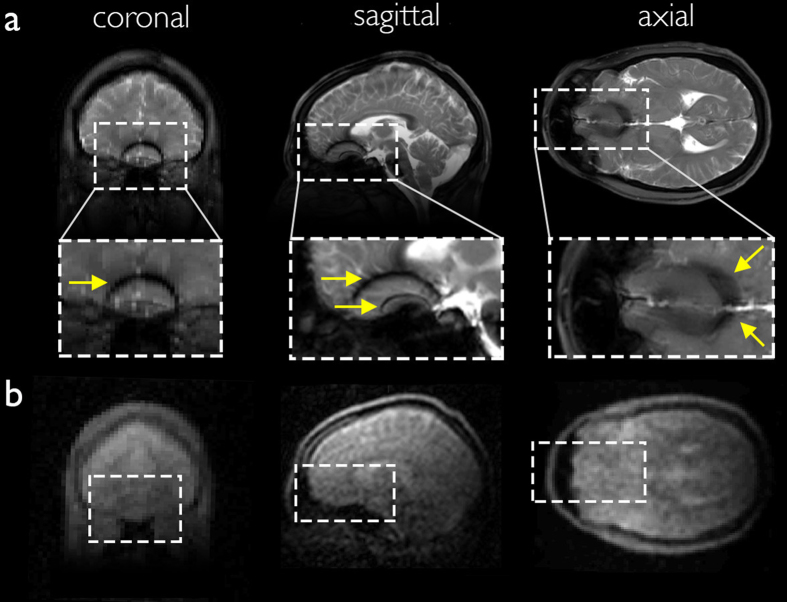
Comparison of b-SFFP images at (**a**) 3T and (**b**) 6.5 mT. Strong banding artifacts appear at high magnetic field (yellow arrows) in all orientations (coronal, sagittal, and axial) whereas no artifact is seen in the images acquired at ultra-low field.

Three-dimensional under-sampled images acquired at 6.5 mT in 6 minutes are shown in Fig. 3 for each of the three spatial orientations (axial, coronal, and sagittal). The maximum image SNR was computed from the ratio of maximum signal amplitude to the standard deviation over a user defined noise region; SNR of 15, 21, and 16 were measured in axial, coronal, and sagittal orientations respectively. With a maximum gradient strength of ~1 mT·m^−1^ and maximal slew rate of 0.7 mT·m^−1^·ms^−1^, no artifacts from concomitant field effect are seen over the 20 cm field of view. The sinuses are easily recognizable in black on the images, as well as the skull. Surrounding the brain, we can identify the dura in bright grey on the coronal and sagittal images (Fig. 3b,c). In the brain, the two hemispheres and the cerebellum are distinct, and cortical tissue can be distinguished from white matter. Liquid compartments, here CSF, appear in bright grey and white. Images acquired in the axial orientation are compared to images acquired in the same subject at high magnetic field (3 T) using traditional 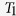, 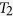, and proton density (*PD*) weighted sequences (Fig. 4). In b-SSFP, contrast is related to the 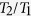 ratio of the imaged sample^25^. At high field, liquids and tissue typically have rather different relaxation times but at 6.5 mT their ratio 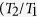) is of order unity resulting in the distinct *PD*-weighted contrast of Fig. 4b. Most of the anatomic features seen at 3 T can be identified in the ultra-low-field scans. Figure 5 compares b-SSFP at 3 T to 0.0065 T.

The maximum SNR in the high field (HF) b-SSFP image is 317. In order to interpret the difference in SNR between the HF and the ULF scans, we scale the HF image SNR by a factor corresponding to the difference in the ULF spatial resolution (2.5 × 3.5 × 8.5 in the axial orientation), and by a second factor to account for signal averaging as done at ULF (Fig. 5b, NA = 160). The SNR in the downscaled HF scan is equivalent to 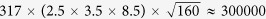, which gives the ratio 
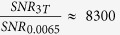
in the axial orientation. If we assume similar coil performance, and neglect the difference in magnetization at steady state for the two magnetic fields, the resulting 8300-fold difference in SNR agrees reasonably well with the simplified approximation that SNR increases with magnetic field to the 3/2 power^42^, here 460^3/2^ = 9866. Strong banding artifacts appear at high field (Fig. 5a, yellow arrows), mainly due to magnetic susceptibility differences at the air-tissue interface. At 276 kHz, on the other hand, no imaging artifact is seen over a 20 cm FOV despite a 3 × longer TR (Fig. 5b). Our results demonstrate excellent immunity to magnetic field inhomogeneity of the order of ±22 Hz, i.e., 160 ppm at 6.5 mT.

## Discussion

The work presented here demonstrates the shortest acquisition times and highest SNR per unit time in ULF MRI to date owing to our use of modern sparse sampling strategies and a fully refocused sequence in an optimized electromagnet scanner. These images were acquired without pre-polarization techniques, at a fixed magnetic field and with a simple single channel inductive detector. With an eye towards optimization, we note that for a given spatial resolution, the minimum TR—and consequently the total scan time—is limited by the maximum attainable time-integrated gradient strength. The maximum gradient strength in the LFI is currently ~1 mT·m^−1^, resulting in a minimum TR of ~23 ms. Weak gradients especially impact phase encoding in balanced sequences like b-SSFP, as every phase-encode pulse is paired with an opposite polarity rewinding pulse. An increase in gradient strength would allow shorter phase encode pulses, thus decreasing total imaging time while maintaining SNR, provided that image distortion from non-linear magnetic fields that accompany the desired encoding gradient (the so called “concomitant field” artifacts^43^) can be mitigated. At 6.5 mT, an increase in gradient strength in the range of 2–5 × , combined with efficient strategies to eliminate concomitant field artifacts^44^,^45^,^46^, can reasonably be envisioned. Additionally, improvements in the electronic noise floor can go a long way to improving scanner sensitivity. In our system, the scanner noise floor is dominated by poor filtering of the high current lines from the gradient power amplifiers into the RF shielded enclosure of the LFI. More effective filtering of electronic noise coming from our gradient power amplifiers would reduce our system noise floor by a measured factor of 3, thus decreasing the total acquisition time by another factor of 3^2^ = 9. As SNR increases with magnetic field to the 3/2 power^42^, a simple doubling of magnetic field would result in a sequence about 8 times faster with similar SNR. In the case of human brain imaging, images with similar resolution and SNR as presented here could be then acquired in less than 3 seconds in such an optimized scanner.

A key challenge in obtaining clinically relevant MRI images at ULF is the ability to acquire *T*_1_ and/or *T*_2_ relaxation-weighted images, and thereby provide contrast to different types of tissue. Typically, magnetization prepared gradient-echo, and spin-echo sequences are used to obtain relaxation-weighted images, but these types of imaging experiments become prohibitively time consuming at ultra-low magnetic fields where signal averaging and recovery of the longitudinal magnetization are required. We have investigated a new strategy to provide contrast based on b-SSFP called “magnetic resonance fingerprinting” (MRF)^47^, and have successfully started its implementation at 6.5 mT.^48^

Finally, theoretical frameworks exist that allow image reconstruction of highly undersampled datasets with multiple channel acquisition^49^,^50^,^51^,^52^,^53^,^54^,^55^,^56^. Acquisition schemes combining high undersampling rates with parallel imaging techniques such as SENSE^57^ or GRAPPA^58^ could reduce the total acquisition time even further, to less than a second. Recent work from Murphy *et al.*^59^ successfully mitigates the computational expense by exploiting massively parallelized computing.

We contend that ULF MRI scanners operating at this expected level of performance could complement traditional MRI by relieving hospital congestion and shortening triage delays. Outside of the radiology suite, mobile ULF scanners might be deployable during military conflicts or during sport events and enable the acquisition of immediate after-trauma knowledge, typically in the case of traumatic brain injuries. Finally, ULF MRI technology may allow resource-poor environments access to MRI systems, without the strict siting requirements and high costs of conventional scanners.

## Methods

### Consent and IRB

Informed consent was obtained from each healthy human volunteer prior to the experiment in accordance with the Human Research Committee of the Massachusetts General Hospital (MGH). All MRI imaging was performed in accordance with approved guidelines and regulations, using experimental protocols that were approved by the MGH Human Research Committee.

### Ultra-low field MRI

All ULF MR images were acquired at 6.5 mT in a healthy human volunteer with the 30-turn single channel well-fitting spiral head coil described above (Fig. 2). The subject was placed supine, head first into the electromagnet. The b-SSFP 3D sequence with under-sampling rate of 50%, acquisition matrix of 64 × 75 × 15, at a spatial resolution of (2.5 × 3.5 × 8.5) mm^3^, (2.5 × 3.5 × 14.4) mm^3^, and (2.5 × 3.5 × 11.5) mm^3^, was used in axial, sagittal and coronal orientations, respectively. TR = 22.5 ms, TE = 11 ms. Total acquisition time was 6 minutes for images with number of averages (NA) = 30 (Fig. 3), and 32 minutes with NA = 160 (Figs 4 and 5). All acquired data were processed using anisotropic diffusion filtering and interpolated in Fourier domain in two dimensions to a 96 × 96 matrix.

### High field MRI

Reference high magnetic field images of the head were acquired in the same subject at 3 T on a standard whole-body scanner (Skyra, Siemens Healthcare) using a 32-channel head receiver coil with the subject in a supine position. All high field sequences were acquired with an acceleration factor of 2. Proton density, *T*_2_, and *T*_1_ weighted sequences were acquired with matrix = 256 × 256 × 176 at a spatial resolution of (1 × 1 × 1) mm^3^, with total acquisition times of 10 minutes, 5 minutes and 6 minutes respectively. A b-SSFP sequence was acquired with matrix = 256 × 256 × 192 at a spatial resolution of (1 × 1 × 1) mm^3^, and NA = 1. The total acquisition time was 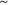
3 minutes.

## Additional Information

**How to cite this article**: Sarracanie, M. *et al.* Low-Cost High-Performance MRI. *Sci. Rep.* **5**, 15177; doi: 10.1038/srep15177 (2015).
